# Special Issue on ‘Asthma and Allergic Inflammation’

**DOI:** 10.3390/vaccines11030531

**Published:** 2023-02-23

**Authors:** Corrado Pelaia

**Affiliations:** Department of Health Sciences, University “Magna Græcia” of Catanzaro, 88100 Catanzaro, Italy; pelaia.corrado@gmail.com

Asthma is a chronic inflammatory airway disease, driven by either allergic or non-allergic stimuli, which usually manifests as wheezing, reversible airflow limitation, and bronchial hyperresponsiveness. The epidemiological impact of asthma is very relevant; indeed, asthmatic people amount to more than 300 million worldwide [1]. Although airway inflammation is the hallmark of asthma pathobiology, this disease is quite heterogenous; thus, it is characterized by several different phenotypes [2]. The latter are underpinned by complex networks of cellular and molecular mechanisms, known as endotypes. The most frequent phenotype/endotype is type 2 (T2) asthma, which is predominantly sustained by bronchial eosinophilic inflammation and can occur in atopic or non-atopic patients [3]. In the first case, asthma is triggered in susceptible individuals by inhaled allergens, including house-dust mite, pollens, animal dander, etc., which penetrate the airway epithelium and activate the Toll-like receptor (TLR) subclass of pattern recognition receptors [4]. TLR stimulation leads to the release from injured airway epithelial cells of innate cytokines such as alarmins, including thymic stromal lymphopoietin (TSLP) and interleukin (IL)-25 and IL-33. Alarmins prime dendritic cells to actively capture, internalize, and process aeroallergens [5]. Upon proteolytic processing, modified inhaled antigens are exposed and presented in regional lymph nodes by dendritic cells to naïve T lymphocytes within the context of human leukocyte antigen (HLA) molecules of the class II major histocompatibility complex (MHC II). Hence, antigens interact with specific T cell receptors (TCR) in the presence of costimulatory molecules (CD80/B7.1, CD86/B7.2, ICOS ligand, OX40 ligand, and their respective counterligands) and high concentrations of IL-4, mostly released by basophils [6]. As a consequence, T helper (Th) cells polarize towards the Th2 lineage and mature Th2 cells are then recruited into the airways, attracted by a chemotactic gradient generated by dendritic cells themselves through the release of the C-C chemokine ligand 17 (CCL17) and C-C chemokine ligand 22 (CCL22) [7]. Within the airways, committed and activated Th2 lymphocytes produce large amounts of Th2 cytokines including IL-4, IL-5, IL-9, and IL-13 [8]. Together with IL-13, IL-4 acts on B cells by promoting immunoglobulin class switching, leading to the synthesis of immunoglobulins E (IgE). IgE bind to their high-affinity (FcεRI) and low-affinity (FcεRII/CD23) receptors expressed by both immune-inflammatory and airway structural cells, thus degranulating mast cells and basophils, facilitating allergen presentation, inhibiting eosinophil apoptosis, and favoring bronchial remodeling [9]. The latter structural response of the airways is also directly stimulated by IL-13. IL-5 is the key cytokine involved in the induction of eosinophil differentiation, activation, chemotaxis, and survival. The IL-5-dependent activation of eosinophils stimulates these cells to secrete proinflammatory and bronchoconstrictive mediators such as cysteinyl leukotrienes (LTC_4_, LTD_4_, and LTE_4_) and prostaglandin D_2_ (PGD_2_), as well as cytotoxic proteins including eosinophil peroxidase, eosinophil cationic protein, eosinophil-derived neurotoxin, and major basic protein, that significantly contributes to further damaging the airway epithelium [10]. Synthesized by Th2-derived Th9 cells, IL-9 acts as a growth factor for mast cells [11]. Moreover, alarmins directly activate the group 2 of innate lymphoid cells (ILC2), thereby inducing these cells to produce a cytokine pattern characterized by a high similarity with the secretory profile of Th2 lymphocytes. Thus, ILC2 and Th2 cells are engaged in a very close interaction between innate and adaptive immune responses that underlie the pathophysiology of allergic asthma. Furthermore, ILC2 are the main immune cells implicated in the development and amplification of non-allergic T2 eosinophilic asthma [12].

A relevant number of asthmatic patients are characterized by T2-low inflammation, especially featured by neutrophilic or mixed (eosinophilic–neutrophilic) cellular infiltration of the airways. Similar to eosinophilic asthma, neutrophilic disease is also dependent on the synergistic cooperation between innate and adaptive immune mechanisms. In fact, both Th17 lymphocytes and group 3 of innate lymphoid cells (ILC3) produce IL-17A and IL-17F, which in turn stimulate immune inflammatory cells (monocytes/macrophages) and resident structural cells (airway epithelial cells and subepithelial fibroblasts) to release powerful neutrophil chemoattractant mediators such as IL-8 (CXCL8) [13]. Other cellular interactions involving innate and adaptive immune responses refer to the bioactivities of Th1 lymphocytes and group 1 of innate lymphoid cells (ILC1), releasing interferon gamma (IFN-γ) and tumor necrosis factor alpha (TNF-α), which contribute to the inflammation of the neutrophilic airway [14]. In addition to actively participating in the pathogenesis of T2-high asthma, the alarmins TSLP and IL-33 appear to also contribute to the development of T2-low disease by eliciting the differentiation of both Th1 and Th17 cells [15]. T2-low neutrophilic asthma is often associated with the most severe disease phenotypes, as well as with the incomplete reversibility of the obstruction of the airway, a smoking habit, respiratory infections, and corticosteroid insensitivity [16,17,18]. A particular subtype of T2-low asthma is caused by infectious agents such as Chlamydia pneumoniae, Mycoplasma pneumoniae, and several viruses. Indeed, infectious asthma is mainly characterized by a neutrophilic inflammatory trait [19,20].

A further phenotype/endotype is paucigranulocytic asthma, which is likely featured by a predominant engagement of airway structural cells such as epithelial cells and especially smooth muscle cells. The latter closely interact with mast cells [21].

The above mentioned cellular and molecular mechanisms underlying airway inflammation in asthma characterize the targets of the currently available anti-asthma therapies. Indeed, the cornerstone of asthma treatment is represented by inhaled corticosteroids, which are able to inhibit the biological activities of several immune-inflammatory cells as well as the production of many cytokines and chemokines [22]. However, various degrees of steroid-insensitivity can occur in severe asthma. Thus, add-on biological therapies of T2-high severe asthma are based on the use of monoclonal antibodies targeting IgE, pro-inflammatory cytokines, or their receptors [23]. The anti-IgE humanized monoclonal antibody omalizumab was the first drug to be introduced for the biological treatment of severe allergic asthma. Mepolizumab and reslizumab are anti-IL-5 humanized monoclonal antibodies. Benralizumab is a humanized monoclonal antibody which selectively blocks the alpha subunit of the IL-5 receptor. Dupilumab is a fully human monoclonal antibody which specifically binds to the alpha subunit of the IL-4 receptor that is utilized by both IL-4 and IL-13, whose bioactivities are thus inhibited by dupilumab at the receptor level. Tezepelumab is a fully human monoclonal antibody which targets TSLP. So far, no biologic drug has been unfortunately developed and approved to treat T2-low severe asthma.

Based on these considerations, the purpose of the present Special Issue is to outline and discuss the recent advances referring to the inflammatory pathways underpinning the phenotypes/endotypes of asthma. Ongoing progress regarding this topic is crucially important to elucidate the cellular and molecular targets of current and future pharmacological treatments of this widespread disease.
